# Testicular Ultrasound Analysis as a Predictive Tool of Ram Sperm Quality

**DOI:** 10.3390/biology11020261

**Published:** 2022-02-08

**Authors:** Melissa Carvajal-Serna, Sara Miguel-Jiménez, Rosaura Pérez-Pe, Adriana Casao

**Affiliations:** Grupo BIOFITER-Departamento de Bioquímica y Biología Molecular y Celular, Instituto Universitario de Investigación en Ciencias Ambientales de Aragón (IUCA), Facultad de Veterinaria, Universidad de Zaragoza, 50013 Zaragoza, Spain; melissac@unizar.es (M.C.-S.); 652325@unizar.es (S.M.-J.); rosperez@unizar.es (R.P.-P.)

**Keywords:** echotexture, ultrasound, Doppler, seasonality, sperm, testes

## Abstract

**Simple Summary:**

In animal production, the prediction of male fertility is vital for the success of specific techniques such as artificial insemination. Thus, testicular ultrasound, a non-invasive diagnostic procedure, could be a useful tool. Moreover, recent ultrasound-video analysis and software developments allow the visualization of tissue at the microscopic level. The objective of this work was to establish a possible correlation between testicular ultrasonography and semen quality in rams. For this purpose, the testicles of nine rams were evaluated and the semen was analyzed for one year. The results revealed that the number of white and grey pixels correlated with sperm parameters indicating poor seminal quality. On the other hand, the increase in the seminiferous-tubule density or the lumen area of these tubules was related to a rise in seminal quality. Therefore, ultrasound-video analysis could be a good tool for evaluating the fertility of rams, either for artificial insemination or on the farm.

**Abstract:**

Testicular ultrasound is a non-invasive technique that could be very useful for predicting ram seminal quality. Recent software developments allow macroscopic and microscopic evaluation of testicular parenchyma. Thus, the aim of this study was to evaluate the testicular echotexture using ultrasound-video analysis and investigate its possible correlation with semen quality. Nine rams were evaluated for one year using a portable ultrasound scanner and the echotexture was analyzed with ECOTEXT^®^ software. The number of black (Ec1), white (Ec2), and grey pixels (Ec3), tubular density (TD), lumen area (LA), and lumen diameter (LD) were analyzed. Semen was collected by an artificial vagina the same day and the sperm concentration, morphology, motility, viability, phosphatidylserine (PS) translocation, reactive-oxygen-species (ROS) levels, DNA damage and capacitation state were evaluated. Ec2 and Ec3 correlated positively with “bad quality” sperm parameters (the percentage of spermatozoa with high ROS levels, with PS translocation and proximal cytoplasmic droplets), and negatively with motility. In contrast, TD and LA showed a positive correlation with “good quality” parameters (motility or normal morphology) and a negative correlation with spermatozoa with high ROS levels, with DNA fragmentation, and proximal or distal cytoplasmic droplets. Thus, echotexture analysis by ultrasound-video analysis could be a valuable tool for assessing ram fertility.

## 1. Introduction

Seasonality, which is regulated by photoperiod and melatonin secretion [1], could be a limiting factor in the sheep reproduction [2]. In the ram, the effect of seasonality is less marked than in the ewe, and some breeds experience variable degrees of seasonality [3,4,5], mainly depending on the latitude in which they are located [6]. The study of seasonality changes in the ram has focused on seminal quality [7,8] or testicular measurement [9,10] rather than changes in testicular parenchyma.

Ultrasonographic evaluation is a valuable, non-invasive technique for diagnosing the male genital tract. It can be used as a complementary test for breeding-soundness evaluation [11]. Additionally, changes in the testis echotexture (pixel intensity and uniformity) due to changes in the tissue composition can be evaluated by ultrasonography and assessed with image-analysis software [12,13] that describes the ultrasound pixel intensity in terms of numerical pixel values (NPV). These range from 0 (absolute black) to 255 (absolute white) and provide an indication of tissue echogenicity [14]. Thereby, ultrasound analysis has been used to predict the reproductive soundness of rams [15,16,17] or the detection of pubertal changes in lambs [18,19]. However, in these previous studies, the ultrasound images were evaluated by non-specific image-analysis software (such as Image ProPlus^®^ or Image J), which could decrease the sensitivity and reproducibility of the results. Recently, a dedicated software package has been specifically developed for studying testicular tissue through macroscopic and microscopic ultrasound-video analysis (Ecotext^®^ software). This software is able to analyze testicle ultrasonograms based on their echotexture properties, measuring pixel intensity as Ec1 (black pixels, the number of pixels with NPV = 0), Ec2 (white pixels, the number of pixels with NPV = 255), and Ec3 (grey level of pixels, the mean value of the pixels with NPV > 0 and <255). It can also evaluate microscopic structures of the testicular parenchyma (up to 200 μm in size) that are usually evaluated by biopsy, such as the seminiferous-tubule (ST) density or the ST lumen area and diameter. This software has already been validated in bulls, rams, stallions, and boars [20,21,22,23].

Testicular parenchyma constitutes a tissue of very high metabolic activity, and it is therefore very sensitive to changes in the blood supply by the testicular artery. Furthermore, testicular blood flow has been correlated with spermatogenesis and spermatozoa fertilization ability in several species [24,25]. Thus, a color-Doppler analysis could provide information about the testicular-artery blood flow and vascular integrity of the male reproductive tract [14]. Color-Doppler has previously been used to evaluate changes in blood-flow velocity and the resistance of the blood in the testicular artery of the ram [19,26] and their relationship with semen quality [27,28].

The main goal of testicular ultrasound, other than to discard males with testicular pathology [29], is to predict the seminal quality and, thus, the fertility of sires. In this context, previous works on the ram have found a moderate correlation between testes blood flow, echotexture and sperm quality [16,27,28], whereas others have not found any correlation [17]. In most of these works, only classical sperm parameters, such as volume, motility, concentration or viability, were evaluated. However, recent studies suggest that these classical sperm parameters have a low correlation with field fertility, and parameters related to sperm physiology, such as oxidative stress, apoptotic levels or capacitation state, should be evaluated for ram-fertility prediction [30,31]. Moreover, spermatogenesis in the ram lasts around sixty days, including epididymal storage. For this reason, the results of a semen evaluation performed at any given time do not reflect the current testicular function and, in the same way, the testicular ultrasound analysis likely relates to future semen quality, but not to the sperm parameters obtained on the day of ultrasound scanning [15].

Hence, the aim of this study was (1) to evaluate seasonal changes in testicular echotexture parameters and testicular blood flow using ultrasound-video analysis and color-Doppler analysis, (2) to investigate the possible correlation between changes in ultrasonographic parameters and semen quality (evaluated with classical and non-conventional sperm-quality parameters) at the moment of scanning (present) and 30 and 60 days later.

## 2. Materials and Methods

Unless otherwise stated, all reagents were purchased from Merck KGaA (Darmstadt, Germany).

### 2.1. Animals and Semen Collection

Semen was collected from nine *Rasa Aragonesa* rams (2–8 years old) belonging to the *Rasa Aragonesa* National Breeding Association (Asociación Nacional de Criadores de Ganado Ovino Selecto de Raza Rasa Aragonesa, ANGRA). The rams were kept under uniform feeding conditions at the University of Zaragoza Veterinary School, Spain (latitude 41°41′ N). All experimental procedures were carried out under the project license PI39/17 approved by the Ethics Committee for Animal Experimentation of the University of Zaragoza (approval date: 24 May 2017), according to the Spanish Policy for Animal Protection RD53/2013, which meets the European Union Directive 2010/63 on the protection of animals used for experimental and other scientific purposes.

The rams were subjected to a regimen of continuous semen extraction throughout the year, with two days of abstinence between collections. For this study, a monthly semen evaluation was performed for one year. Second ejaculates from each male were collected individually using an artificial vagina, and semen was maintained at 37 °C until laboratory analysis. Semen samples were diluted 1/100 in a medium containing 0.25 mol/L sucrose, 10 mmol/L Hepes, 2 mmol/L KOH, 5 mmol/L glucose, 0.5 mol/L NaH_2_PO_4_ and 100 mmol/L EGTA for assessing sperm quality. Sperm concentration was calculated in duplicate using a Neubauer chamber (Marienfeld, Lauda-Konigsofen, Germany).

### 2.2. Sperm Motility Evaluation

Motility parameters were measured using a computer-assisted CASA system (ISAS 1.0.4; Proiser SL, Valencia, Spain). Sperm motility was recorded utilizing a video camera (Basler A312f, Basler AG, Ahrensburg, Germany) mounted on a microscope (Nikon Eclipse 50i, Nikon Instruments Inc, Tokyo, Japan) equipped with a 10x negative-phase contrast lens and a 10x ocular lens. Samples (6 µL of the 1/100 semen dilution) were placed between pre-warmed slides and coverslips and kept at 37 °C in a heated slide holder during analysis. For each sample, five videos at 25 frames/second for 1 s were recorded. The percentages of motile (TM) and progressive motile (PM) spermatozoa were evaluated.

### 2.3. Flow Cytometry Analysis

All the sperm cytometry analyses were performed on a Beckman Coulter FC 500 flow cytometer with CXP software (Beckman Coulter Inc., Brea, CA, USA) equipped with two excitation lasers (Argon-ion laser 488 nm and Red solid-state laser 633 nm) and five absorbance filters (FL1–525, FL2–575, FL3–610, FL4–675 and FL5–755 ± 5 nm each bandpass filter). A minimum of 20,000 events were evaluated in all the experiments. The sperm population was identified for further analysis based on their specific-forward (FS) and side-scatter (SS) properties; thus, other non-sperm events were excluded. A flow rate stabilized at 200–300 cells/sec was used.

#### 2.3.1. Sperm Viability

Three microliters of carboxyfluorescein diacetate (CFDA, 1 mM), 3 µL of propidium iodide (PI, 0.75 mM) and 5 µL of formaldehyde (0.5% *v*/*v* in water) were added to 500 µL of sperm samples (6 × 10^6^ cells/mL) based on a modification of the method described by Harrison and Vickers [32]. After 15 min at 37 °C in the dark, the samples were analyzed by flow cytometry. The Argon-ion laser and the 525 (FL1, CFDA) and 675 nm (FL4, PI) filters were used in order to avoid overlapping. The percentage of CFDA+/PI- spermatozoa (viable cells) was evaluated.

#### 2.3.2. Intracellular Reactive Oxygen Species (ROS)

Aliquots of samples (500 µL), prepared at a final concentration of 6 × 10^6^ cells/mL, were stained with 5 µL of H_2_DCFDA (20 mM) and PI (1.5 mM). After 15 min at 37 °C in the dark, the samples were fixed with 5 µL formaldehyde (0.5% *v*/*v* in water) and analyzed by flow cytometry [33]. The Argon-ion laser and 525 and 675 nm filters were used in order to avoid overlapping. The parameters monitored were FS log, SS log, FL1 (H_2_DCFDA), and FL4 (PI). The percentages of viable spermatozoa with low ROS levels and spermatozoa with high ROS levels were evaluated.

#### 2.3.3. Detection of Membrane Phosphatidylserine (PS) Translocation

Annexin V is a calcium-dependent phospholipid-binding protein with a high affinity for PS. FITC-Annexin V (Thermo Fisher Scientific, Waltham, MA, USA) was used simultaneously with PI to detect PS translocation and to differentiate between membrane-intact and damaged cells, with or without PS translocation. Aliquots of 300 µL (4 × 10^6^ cells diluted in binding buffer) were stained with FITC-Annexin V (2 µL) in combination with 7.5 µM PI (3 µL), incubated at 37 °C in the dark for 15 min, and evaluated by flow cytometry. The monitored parameters were FS log, SS log, FL1 (FITC-Annexin V), and FL4 (PI). The percentages of viable spermatozoa without PS translocation (non-apoptotic, Annx-/PI-), and spermatozoa with PS translocation (Annx+) were considered.

#### 2.3.4. DNA Fragmentation—TUNEL Assay

The presence of DNA strand breaks in ram spermatozoa was assessed using the TUNEL assay with fluorescein-isothiocyanate (FITC)-labeled dUTP (In Situ Cell-Death-Detection Kit)). Sperm samples (4 × 10^7^ cells/mL) were fixed with 4% (*w*/*v*) paraformaldehyde in PBS at room temperature (RT) for 1 h. After two washes at 600× *g* with 100 µL PBS, the samples were permeabilized with 0.1% Triton X-100 (*v*/*v*) in 0.1% sodium citrate (*w*/*v*). After centrifugation at 600× *g*, the pellet obtained was incubated with 50 µL of labelling solution containing the TdT enzyme and dUTP for 1 h at 37 °C in the dark. A negative control was prepared for each experimental set by omitting TdT from the reaction mixture. After two consecutive washes with PBS at 600× *g* for 10 min at RT to stop the reaction, flow-cytometry analysis was performed. The monitored parameters were FS log, SS log, and FL1 (TUNEL). The percentage of sperm with DNA fragmentation (TUNEL+ cells) was evaluated.

### 2.4. Assessment of Capacitation Status by CTC Staining

The capacitation status was determined using chlortetracycline (CTC) staining [34]. A CTC solution (750 μM) was prepared daily in a buffer containing 20 mM Tris, 130 mM NaCl and 5 μM cysteine (pH 7.8) and passed through a 0.22 μm filter. After that, 20 μL of CTC solution and 5 μL of 12.2% (*w*/*v*) paraformaldehyde in 0.5 M Tris–HCl (pH 7.8) were added to a 20 μL sperm sample (4 × 10^7^ cells/mL) and incubated at 4 °C in the dark for at least 30 min. At room temperature and semi-darkness, a 4 μL aliquot of the stained sample was placed on a glass slide and mixed with 2 μL of 0.22 M triethylenediamine (DABCO) in glycerol:PBS (9:1, *v*/*v*). Samples were covered with 24 × 48 mm coverslips, sealed with colorless enamel, and stored in the dark at −20 °C. To evaluate CTC patterns, samples were examined using a Nikon Eclipse E-400 microscope (Kanagawa, Japan) under epifluorescence illumination using a V-2A filter. All samples were processed in duplicate and 200 spermatozoa were scored per slide. Sperm classification followed Gillan et al. [35]: non-capacitated spermatozoa (NC with even yellow fluorescence over the head, with or without a bright equatorial band); capacitated cells (C, with fluorescence on the acrosome) and acrosome-reacted cells (R, without fluorescence on the head and with or without a bright equatorial band).

### 2.5. Morphological Study by Eosin-Nigrosine Staining

Semen samples (20 µL of 4 × 10^7^ cells/mL) were mixed with 10 µL eosin and 10 µL nigrosine. One droplet of 20 µL of the stained sample was smeared onto a clean slide with the help of another slide. The smears were air-dried and examined by bright-field microscopy at 1000X magnification using a Nikon Eclipse E-400 microscope (Kanagawa, Japan). At least 200 spermatozoa were analyzed. The percentage of cells with normal morphology and abnormal spermatozoa, including primary (detached head) and secondary (proximal or distal droplet, bent tail and coiled tail) abnormalities, were evaluated [36].

### 2.6. Testes Measurement and Ultrasonography Examination

Scrotal circumference (SC) was measured using a measuring tape positioned around the scrotum’s largest circumference. Testis length (TL) and width (TW) were measured using a caliper. TL was measured from the head of the epididymis to the top of the tail of each testis, whereas TW was evaluated in the widest part of the testis. Testicular volume (TV) was determined using the equation proposed by Godfrey et al. [37]: TV = 0.0396 × average testis length of both testes (TL) × SC^2^.

The ultrasonography evaluation of the testis was carried out using a portable ultrasound scanner (ExaGo, IMV imaging Angoulême, France) connected to a 7.5 MHz linear probe. The same researcher performed all evaluations, and the ultrasound scanner parameters were adjusted to 60 mm depth, 100% power, 0 dB gain and 60 dB dynamic range. The rams were restrained and no sedatives were used. The probe was positioned transversely to major axis of the testicle. Three videos of 124 frames each were recorded in the upper, medium and lower parts of each testicle for echotexture analyses.

The echotexture analysis was performed with ECOTEXT^®^ software (Humeco, Huesca, Spain). Three testicular parenchymatic characteristics were evaluated at standard resolution: the number of black (ECOTEXT 1, Ec1, referring to the number of pixels with a numerical pixel value of 0), white (ECOTEXT 2, Ec2, referring to the number of pixels with a numerical pixel value of 255), and grey pixels (ECOTEXT 3, Ec3, mean value of the pixels with a numerical pixel value > 0 and <255); another three were evaluated at high resolution: the density of tubules/cm^2^ (tubular density, TD), the percentage (%) of the total area occupied by the lumen of the tubules in the parenchyma (lumen area, LA), and the mean diameter (μm) of the lumen of the seminiferous tubules (lumen diameter, LD).

Color-Doppler flow imaging was used to analyze the arterial blood flow of the pampiniform plexus. All color-Doppler scans were performed with a constant gain (20 dB), 50 Hz high pass filter, and 1 mm gate setting (Hedia et al., 2019). The angle between the Doppler beam and each vessel’s long axis was ≤60, and the pulse-repetition frequency (2,000 Hz) was adjusted to reduce aliasing. After the spectral pattern of the testicular artery was generated, the frequency (bpm), the resistive index (RI = (maximum velocity−minimum velocity)/maximum velocity) and the pulsatility index (PI = (maximum velocity−minimum velocity)/mean velocity) were calculated [38]. Five measurements were taken along the path of the artery for each testis, and at least five waveforms were recorded per measurement.

### 2.7. Statistics Analyzes

Monthly and seasonal results are shown as mean ± SEM of the number of samples assessed in each case. Data distribution was analyzed by the Kolmogorov–Smirnov test, and outliers were identified by the Grubbs test. The difference between breeding (B: August to February) and non-breeding seasons (NB: March to July) in terms of concentration and progressive motility, TW, Ec3, TD, LD, frequency and RI were analyzed by the unpaired *t*-test. The rest of the parameters were assessed by the Mann–Whitney test. Differences between the right and left testicles were evaluated by the paired *t*-test for width, Ec3, TD, LD, frequency and RI, and the Wilcoxon matched-pairs signed-rank test for their length, Ec1, Ec2, LA and PI. The correlations between the ultrasound results and the sperm parameters, which were obtained on the same day, 30 and 60 days after the testicular scanning, were evaluated by Spearman’s test. All statistical analyses were performed using SPSS (v.15.0) software.

## 3. Results

In this study, nine rams were subjected to semen collection and sperm-quality evaluation, followed by an ultrasonography examination of the testes every month for one year. All the rams showed suitable sperm quality after evaluation, but one showed calcification spots during the ultrasonography examination; hence, that male was eliminated from the study.

### 3.1. Changes in Size and Ultrasound Evaluation of the Testes and Ram Sperm Quality between Seasons

When the left and right testes were compared, only the testicular length, tubular density (evaluated by ultrasonography), and frequency (evaluated by Doppler analysis) showed significant differences (*p* < 0.05, Supplemental Table S1). Thus, the mean values of the right and left testicles were used for further evaluation.

No differences were found between the breeding (B) and non-breeding (NB) seasons in terms of testis size (Table 1), although an increase in testicular length and volume during the hottest months of the year can be observed (Supplemental Figure S1A). However, regarding the scanning results, we found seasonal differences (*p* < 0.05) in Ec1 and the seminiferous-tubule lumen diameter (LD), which increased during the B season (Table 1). The rest of the echogenicity parameters did not show any changes. Of the Doppler parameters, only the frequency significantly increased (*p* < 0.05) during the non-breeding season (Table 1), although a non-significant decrease in PI was observed in the coldest months of the year (Supplemental Figure S1B).

**Figure 1:**
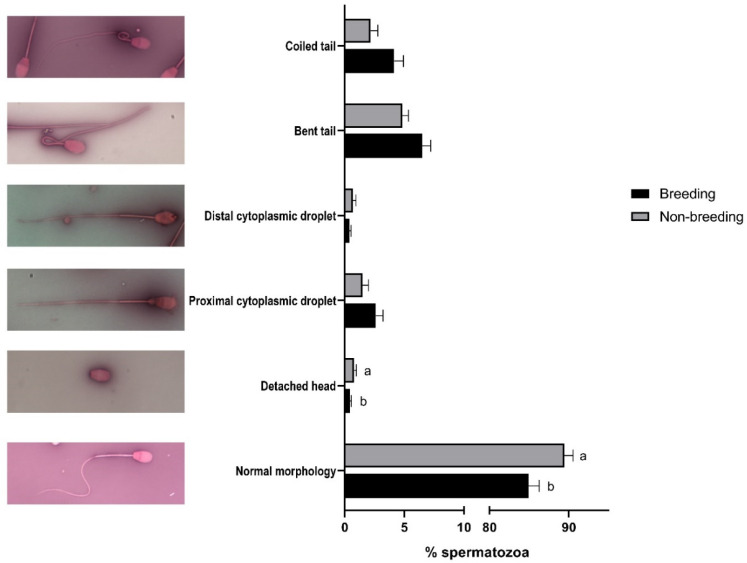
Seasonal differences in sperm morphology in *Rasa Aragonesa* rams. Results are shown as mean ± SEM of *n* = 56 (breeding season) and *n* = 40 (non-breeding season). Different letters indicate *p* < 0.05. Representative images of a normal spermatozoa and sperm abnormalities are also shown.

**Figure 2:**
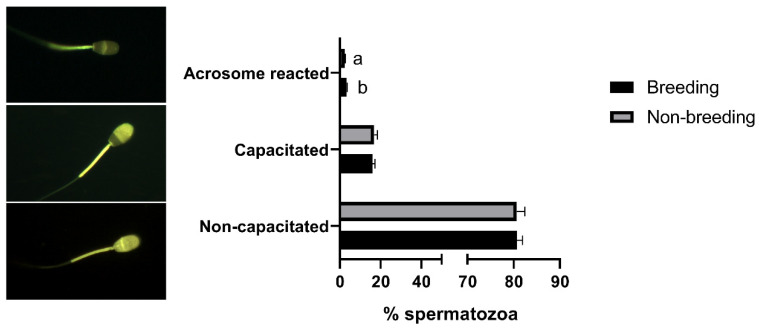
Seasonal differences in sperm capacitation state in *Rasa Aragonesa* rams. Results are shown as mean ± SEM of *n* = 56 (breeding season) and *n* = 40 (non-breeding season). Different letters indicate *p* < 0.05. Representative images of a non-capacitated, capacitated and acrosome-reacted spermatozoa are also shown.

When sperm quality was evaluated, significant differences (*p* < 0.05) between the breeding and non-breeding seasons were observed in the percentages of spermatozoa with high ROS levels (Table 2), with normal morphology, with a detached head (Figure 1), and in the rate of acrosome-reacted cells (Figure 2). Nonetheless, when differences between months were evaluated, significant differences were observed in nearly all the studied parameters, being more marked between March–April and September (Supplemental Figure S2).

### 3.2. Correlation between Testicular Parameters and Sperm-Quality Values

We evaluated the correlation between the testicular parameters (testes measurement and ultrasound analysis) and the sperm-quality parameters at the moment of the testis evaluation, and at 30 days or 60 days afterwards. Most correlations were found when the sperm parameters were evaluated simultaneously (Table 3) or 30 days after (Table 4) the testicular analysis. However, most of these relationships were not apparent when sperm quality was evaluated 60 days afterwards (Table 5).

When the correlations between testicular size and sperm parameters were evaluated, the most significant result was a negative one with the viable spermatozoa without PS translocation, which was found during the three periods of time analyzed (0, 30 or 60 days later; Table 3, Table 4 and Table 5). We also found a negative correlation between testicular length and viability after 30 and 60 days of the measurement and viable spermatozoa with low ROS levels after 60 days (Table 4 and Table 5). A positive correlation between scrotal circumference and normal sperm morphology was also found after 30 days (Table 3). Testicular size (length or width) correlated negatively with sperm abnormalities (proximal and distal cytoplasmic droplet or bent tail) the same day and 30 days later (Table 3 and Table 4), although testicular length also correlated positively with the percentage of spermatozoa with a detached head (Table 4).

Regarding the echotexture parameters, the macroscopic (standard resolution Ec1-black pixels, Ec2-white pixels and Ec3-grey pixels) correlated mainly with sperm parameters that were evaluated 30 days after the scanning (Table 4). When the analysis was performed on the same-day or 60-day sperm parameters, only a correlation between Ec2 and spermatozoa with high ROS levels (Table 3) or progressive motility (Table 5) was found. In general, Ec2 and Ec3 correlated positively with “bad quality” sperm parameters (the percentage of spermatozoa with high ROS levels (Table 3) or with PS translocation and proximal cytoplasmic droplet (Table 4)), and negatively with “good quality” sperm parameters (total and progressive motility, Table 4 and Table 5, respectively). Ec1 only correlated negatively with the percentage of spermatozoa with a detached head 30 days after scanning.

Among the microscopic, high-resolution echotexture parameters, the tubular density (TD) showed more correlations with the sperm parameters. Overall, TD showed a positive correlation with “good quality” sperm parameters, such as total (Table 3) and progressive motility (Table 3 and Table 4), and normal morphology (Table 3), and a negative correlation with the “bad quality” sperm parameters: the percentage of spermatozoa with high ROS levels (Table 3), with DNA fragmentation (Table 4) or with proximal (Table 3 and Table 4) or distal cytoplasmic droplet (Table 3, Table 4 and Table 5). However, a negative correlation between TD and sperm concentration (Table 3 and Table 5) or viability (Table 4 and Table 5) was also found. The lumen area correlated negatively with total spermatozoa with high ROS levels that were obtained the same day of scanning and positively with the total motility of the sample obtained 30 days later. Lumen diameter did not correlate with any sperm parameters.

The main correlations for the Doppler parameters were found with sperm parameters that were evaluated the same day of the ultrasound analysis (Table 3). The frequency correlated positively with sperm concentration and the percentage of spermatozoa with a bent tail and negatively with total motility, whereas PI and RI correlated negatively with sperm concentration and spermatozoa with high ROS levels. PI was also positively related to total motility. These correlations were not apparent when the semen quality was studied 30 days after Doppler evaluation, and only the frequency–sperm-concentration and RI–high-ROS-level correlations were maintained (Table 4). Finally, a negative correlation between the frequency and non-capacitated spermatozoa and a positive correlation with capacitated sperm were found when the sperm parameters were evaluated after 60 days (Table 5).

### 3.3. Correlation between Ultrasonographic and Morphometric Parameters

All testicular-measurement parameters correlated negatively with Ec1, LA and LD and positively with Ec3 (except for testicular width, which showed a negative correlation with LD). Ec2 correlated positively with testicular volume and scrotal circumference (Table 6). No correlation was found between testicular measures and tubular density.

On the other hand, when the correlation between the Doppler parameters and echotexture was evaluated, the frequency showed a negative correlation with Ec1 and LA (Table 7). PI and RI correlated negatively with Ec2 and Ec3 and positively with Ec1, TD, LA and LD. No correlation was found between the Doppler parameters and testicular size (Table 7).

## 4. Discussion

Testicular ultrasonography has been postulated, along with testes measurement, as a non-invasive method to assess the reproductive soundness of sires. Moreover, recent software and ultrasound-probe developments have allowed the evaluation of microscopic structures within the testis parenchyma. Thus, in this work, we have investigated seasonal variations in testicular echotexture parameters, evaluated at the macroscopic and microscopic level by ultrasound-video analysis, and their relationship with testicular size and sperm-quality parameters.

The mean values obtained after echotexture evaluation with ECOTEXT^®^ software were similar to those previously reported for the ram [39]. When the effect of the season was evaluated, a decrease in Ec1 (black pixels) and the seminiferous-tubule lumen diameter (LD) during the non-breeding season were found. Previous studies had shown an increase in testicular numerical-pixel values (NPVs) and pixel heterogeneity during the NB season [15,16] that could have been related to changes in the seminiferous tubules [13,14]. Our work revealed that these seasonal differences were due to a decrease in the number of black pixels and the diameter of the ST lumen, without changes in ST density or the percentage of the lumen area. Regarding the Doppler analysis, we only found seasonal differences in pulse frequency, in contrast with Hedia et al. [27], who recorded a marked increase in RI and PI values during the spring and summer. However, despite the lack of seasonal differences, we similarly detected significant differences in PI between the coldest and the hottest months of the year. Thus, the changes in Doppler parameters in the ram could be due to environmental temperature changes and not to physiological variations due to seasonal reproduction, as has been suggested for bucks [26].

We also studied testicular size and volume throughout the year because they are commonly considered a good index of sperm production [40] and an important feature in male selection [41]. Although previous studies suggested a seasonal variation in ram testicular size and volume [4,8,42], our study showed no significant differences in any measurements analyzed between B and NB seasons. However, we found some differences between the hottest and coldest months of the year, as in other sheep breeds [27,43]. Moreover, in other studies, a significant positive correlation between testicle volume and air temperature has also been observed [40,43].

When the correlation between testicular measurement and ultrasound values was evaluated, we found a negative correlation between testis size and volume and Ec1 and a positive correlation with Ec2 and Ec3. Thus, as testicular size increases, black pixels decrease, and white and grey pixels increase. This result suggests that the testicular size increase is caused by the rise in the number of cells within the seminiferous tubules and not by an increase in tubule density or lumen size. This hypothesis is corroborated by the negative correlation between testicular size and lumen-related parameters (lumen area and diameter) and the lack of correlation with tubular density. Previous studies showed that testicular lipid-content variations could be evaluated by testicular ultrasonography [44]. Lipids are highly echogenic, so increasing cellular density would increase the grey and white pixels due to the lipids present in the cell membranes. Other studies have also demonstrated that the increase in the testes echogenicity during puberty in bull calves was due to a rise in the numbers of cells within the seminiferous tubules [45].

We did not find any correlation between testicular measurements and the Doppler parameters, in contrast with previous studies [27] in which a negative correlation between testicular volume and RI and PI was found. However, the method used in those studies for the testicular-volume calculation was different. Nonetheless, when the correlation between echotexture and the Doppler parameters was evaluated, PI and RI correlated negatively with Ec2 and Ec3 and positively with Ec1 and all of the microscopic ultrasound parameters. RI and PI are inversely linked to the blood-flow perfusion, so our results suggest that an increase in blood flow in the testis would increase the number of cells within the seminal tubule, thus reducing the lumen area as other authors suggested [45].

Nonetheless, the main goal of our study was to determine the predictive capacity of testicular echography (both echotexture evaluation and testicular-artery Doppler analysis) and testicular measurement on sperm quality, evaluated with classical and non-conventional parameters.

We only found seasonal differences in the percentage of spermatozoa with high ROS levels, acrosome-reacted spermatozoa and sperm morphology. These results contrast with other studies, which found differences in motility, concentration, sperm morphology or membrane integrity [7,16,46]. It is probable that our continuous regime of semen extraction can overcome the loss of seminal quality provoked by the non-reproductive season in the ram, although differences in breed or geographical location could also influence the response to seasonality [47].

The correlation analysis was performed between ultrasound parameters and seminal quality at the moment of scanning and 30 and 60 days later. Most correlations were found with semen that was obtained the same day or 30 days after ultrasonography. Ram spermatogenesis in the seminiferous tubule lasts 47 days [48]. ECOTEXT^®^ software evaluates ST density and lumen characteristics, which could be more closely related to the final phase of the spermatogenesis, i.e., the spermiogenesis. Other similar studies using the pixel intensity and pixel heterogeneity of testicular parenchyma had observed correlations between testicular echotexture and the quality of semen obtained two or four weeks after ultrasound examination in bulls [49], and the same day [16] or 60 days afterwards in rams [15]. This suggests that testicular pixel values might be a good predictor of future sperm quality. Our study, in which we could differentiate between black, white and grey pixels, revealed that Ec2 and Ec3 (white and grey pixels, respectively) correlated positively with “bad quality” sperm parameters and negatively with “good quality” ones, especially between those parameters related to apoptosis and the sperm collected 30 days after ultrasound evaluation. Thus, an increase in Ec2 and Ec3 could indicate a present or near-future decrease in seminal quality. In this study, we also found that the increase in testicular size increased Ec2 and Ec3, and a negative correlation between testicular dimensions and viability, viable sperm without PS translocation, and low ROS levels. Thus, if an increase in white and grey pixels indicates an increase in the number of cells within the seminiferous tubules, as studies in prepubertal males suggest [13,45,50], then this excess of cells could initiate the apoptosis process or induce oxidative stress that could compromise future seminal quality and fertility [31,51]. Regarding microscopic echotexture parameters, TD and LA correlated positively with “good quality” sperm parameters and negatively with “bad” ones, especially with those related to sperm morphology and ROS or apoptotic damage, which suggests that both of them could be predictors of good seminal quality. Although other studies have previously observed a correlation between testicular echotexture and sperm morphology [15,16] or DNA fragmentation [28], this is the first time, to the best of our knowledge, that testicular echotexture has been correlated to sperm physiological parameters such as oxidative stress or apoptotic state.

Finally, when the Doppler results were analyzed, the majority of correlations were found with seminal-quality parameters that were evaluated the same day. We found a negative correlation between both PI and RI with sperm concentration, consistent with the results obtained by Hedia et al. [27] and Ntemka et al. [28], but also with spermatozoa with high ROS levels. Thus, although the semi-hypoxic environment in the testis could prevent oxygen-radical damage to sperm [52], changes in blood-flow perfusion and oxygen tension could affect sperm quality at the moment of ejaculation or even a month later [53].

## 5. Conclusions

In conclusion, echotexture analysis by ultrasound-video analysis could be a valuable tool for assessing the breeding soundness of rams. An increase in Ec2 and Ec3 could indicate a decrease in seminal quality, and tubular density and lumen area could be predictors of good seminal quality.

## Figures and Tables

**Table 1:** Seasonal differences in testicular size, echotexture parameters (evaluated by ultrasound-video analysis and ECOTEXT^®^ software) and testicular-artery blood flow (assessed by color-Doppler flow imaging) in *Rasa Aragonesa* rams. Results are shown as mean ± SEM. Different letters indicate *p* < 0.05.

	Breeding Season (*n* = 56)	Non-Breeding Season (*n* = 40)
**Testicular size**		
Scrotal circumference (cm)	35.1 ± 0.4	36.21 ± 0.39
Testicular width (cm)	7.09 ± 0.08	7.21 ± 0.08
Testicular length (cm)	10.54 ± 0.19	10.69 ± 0.2
Testicular volume (cm3)	524.43 ± 18.6	563.3 ± 21.42
**ECOTEXT^®^ parameters**		
Ec1 (black pixels)	23.24 ± 2.46 ^a^	14.03 ± 1.88 ^b^
Ec2 (white pixels)	166.21 ± 19.7	187.29 ± 27.96
Ec3 (grey pixels)	100.96 ± 0.97	103.1 ± 1.3
TD (density of tubules/cm3)	147.97 ± 2.11	151.25 ± 1.91
LA (lumen area, %)	8.54 ± 0.3	7.71 ± 0.32
LD (lumen diameter, μm)	103.68 ± 1.49 ^a^	97.67 ± 1.58 ^b^
**Doppler parameters**		
Frequency (bpm)	90.02 ± 2.51 ^a^	96.69 ± 2.06 ^b^
RI (Resistive index)	0.56 ± 0.02	0.55 ± 0.02
PI (Pulsatility index)	0.89 ± 0.04	0.84 ± 0.05

**Table 2:** Seasonal differences in seminal quality parameters in *Rasa Aragonesa* rams. Results are shown as mean ± SEM. Different letters indicate *p* < 0.05.

Sperm Parameter	Breeding Season (*n* = 56)	Non-Breeding Season (*n* = 40)
Sperm concentration (×10^6^ cells/mL)	4203.36 ± 176.74	4561.00 ± 237.38
Total motility (%)	90.36 ± 0.69	91.28 ± 0.66
Progressive motility (%)	41.66 ± 1.24	44.95 ± 1.52
Membrane integrity (Viability %)	75.99 ± 1.44	80.37 ± 1.15
Viable spermatozoa without PS ^1^ translocation (%)	62.17 ± 2.17	59.98 ± 2.27
Spermatozoa with PS ^1^ translocation (%)	15.23 ± 1.12	16.85 ± 1.13
Viable spermatozoa with low ROS ^2^ levels (%)	73.3 ± 1.3	74.55 ± 0.97
Spermatozoa with high ROS ^2^ levels (%)	7.73 ± 0.35 ^a^	9.79 ± 0.4 ^b^
Sperm with DNA fragmentation (%)	8.55 ± 0.46	8.78 ± 0.52

^1^ PS: phosphatidylserine. ^2^ ROS: reactive oxygen species.

**Table 3:** Spearman’s rank correlation coefficient (Spearman’s ρ) between testicular size, echotexture parameters (evaluated by ultrasound-video analysis and ECOTEXT^®^ software) or testicular-artery blood flow (assessed by color-Doppler flow imaging) and some sperm parameters, analyzed the same day of the testes evaluation, in *Rasa Aragonesa* rams (*n* = 96). Bold type indicates statistically significant correlations. * *p* < 0.05 and ** *p* < 0.01.

	Sperm Concentration	Total Motility	Progressive Motility	Viable Spermatozoa without PS Translocation	Total Spermatozoa with High ROS Levels	Normal Morphology	Proximal Cytoplasmic Droplet	Distal Cytoplasmic Droplet	Bent Tail
**Testicular size**									
Scrotal circumference	−0.141	0.186	0.042	−0.011	0.190	0.177	0.027	−0.138	−0.166
Testicular width	−0.300 **	0.124	0.094	0.012	0.148	0.138	−0.139	−0.163	−0.239 *
Testicular length	−0.162	0.025	−0.017	−0.265 **	−0.024	0.172	−0.234 *	0.007	0.047
Testicular volume	−0.173	0.060	0.138	−0.158	0.107	0.190	−0.082	−0.092	−0.091
**ECOTEXT^®^ parameters**									
Ec1 (black pixels)	−0.032	0.17	0.101	0.070	−0.175	0.051	−0.049	−0.186	0.139
Ec2 (white pixels)	0.168	−0.171	−0.127	−0.108	0.201 *	−0.116	0.175	0.037	−0.036
Ec3 (grey pixels)	0.142	−0.17	−0.081	−0.114	0.184	−0.079	0.12	0.004	−0.064
TD (tubular density)	−0.218 *	0.2	0.371 **	−0.030	−0.225 *	0.233*	−0.338 **	−0.263 **	0.094
LA (lumen area)	−0.101	0.158	0.182	0.133	−0.238*	0.109	−0.167	−0.144	0.155
LD (lumen diameter)	−0.024	0.122	0.027	0.173	−0.182	0.024	−0.006	−0.049	0.123
**Doppler parameters**									
Frequency	0.203 *	−0.210 *	−0.078	0.072	0.107	−0.196	−0.005	0.05	0.283 **
PI (Pulsatility index)	−0.310 **	0.203 *	0.054	−0.004	−0.304 **	0.019	−0.176	−0.028	−0.009
RI (Resistive index)	−0.30 3**	0.186	0.076	−0.012	−0.317 **	0.045	−0.198	−0.043	−0.002

**Table 4:** Spearman’s rank correlation coefficient (Spearman’s ρ) between testicular size, echotexture parameters (evaluated by ultrasound-video analysis and ECOTEXT^®^ software) or testicular-artery blood flow (assessed by color-Doppler flow imaging) and some sperm parameters, analyzed 30 days after the testes evaluation, in *Rasa Aragonesa* rams (*n* = 96). Bold type indicates statistically significant correlations. * *p* < 0.05 and ** *p* < 0.01.

	Sperm Concentration	Total Motility	Progressive Motility	Viability	Viable Spermatozoa without PS Translocation	Total Spermatozoa with PS Translocation	Total Spermatozoa with High ROS Levels	Sperm with DNA Fragmentation	Normal Morphology	DETACHED HEAD	Proximal Cytoplasmic Droplet	Distal Cytoplasmic Droplet
**Testicular size**												
Scrotal circunference	−0.140	0.080	0.039	−0.056	−0.087	0.074	0.129	0.093	0.203 *	−0.003	−0.029	−0.129
Testicular width	−0.167	0.205 *	0.007	0.009	−0.062	0.039	0.080	−0.034	0.196	0.032	−0.024	−0.212 *
Testicular length	−0.195	−0.161	−0.065	−0.229 *	−0.284 *	0.081	−0.070	0.115	0.105	0.255 *	−0.082	−0.042
Testicular volume	−0.172	−0.026	0.003	−0.162	−0.213 *	0.097	0.046	0.122	0.167	0.110	−0.041	−0.098
**ECOTEXT^®^ parameters**												
Ec1 (black pixels)	−0.015	0.14	−0.016	−0.052	0.090	−0.099	−0.099	−0.15	−0.140	−0.251 *	−0.065	−0.174
Ec2 (white pixels)	−0.013	−0.255 *	−0.124	0.001	−0.071	0.236 *	0.169	0.137	0.044	0.039	0.201 *	0.132
Ec3 (grey pixels)	−0.014	−0.232 *	−0.086	−0.034	−0.104	0.218 *	0.150	0.12	0.108	0.091	0.147	0.127
TD (tubular density)	−0.134	0.234 *	0.295 **	−0.226 *	−0.052	−0.145	−0.194	−0.216 *	0.171	−0.087	−0.291 **	−0.312 **
LA (lumen area)	−0.043	0.229 *	0.125	−0.081	0.098	−0.194	−0.140	−0.17	−0.079	−0.157	−0.134	−0.207 *
LD (lumen diameter)	0.038	0.157	−0.015	0.05	0.130	−0.138	−0.098	−0.102	−0.153	−0.156	−0.105	−0.09
**Doppler parameters**												
Frequency	0.232 *	0.091	0.023	0.029	0.083	−0.039	0.050	0.04	0.062	−0.021	−0.095	−0.054
PI (Pulsatility index)	−0.164	0.071	0.032	−0.159	−0.067	−0.001	−0.194	0.021	0.046	0.057	−0.149	−0.101
RI (Resistive index)	−0.179	0.068	0.027	−0.168	−0.081	−0.001	−0.206 *	0.008	0.086	0.074	−0.184	−0.12

**Table 5:** Spearman’s rank correlation coefficient (Spearman’s ρ) between testicular size, echotexture parameters (evaluated by ultrasound-video analysis and ECOTEXT^®^ software) or testicular-artery blood flow (assessed by color-Doppler flow imaging) and some sperm parameters, analyzed 60 days after the testes evaluation, in *Rasa Aragonesa* rams (*n* = 96). Bold type indicates statistically significant correlations. * *p* < 0.05 and ** *p* < 0.01.

	Sperm Concentration	Progressive Motility	Viability	Viable Spermatozoa without PS Translocation	Viable Spermatozoa with low ROS Levels	Non-Capacitated Sperm	Capacitated Sperm	Distal Cytoplasmic Droplet
**Testicular size**								
Scrotal circunference	−0.105	−0.030	−0.072	−0.063	−0.156	−0.019	−0.008	−0.156
Testicular width	−0.155	−0.093	0.011	−0.081	−0.057	−0.005	−0.024	−0.056
Testicular length	−0.139	−0.172	−0.281 **	−0.344 **	−0.235 **	−0.042	0.020	−0.053
Testicular volume	−0.124	−0.090	−0.191	−0.223 *	−0.221 *	−0.022	−0.009	−0.127
**ECOTEXT^®^ parameters**								
Ec1 (black pixels)	−0.043	0.09	0.087	0.095	0.053	0.063	−0.09	−0.026
Ec2 (white pixels)	−0.052	−0.231 *	0.03	0.033	−0.051	0.02	0.014	0.116
Ec3 (grey pixels)	−0.099	−0.178	−0.019	−0.055	−0.083	0.026	0.016	0.066
TD (tubular density)	−0.329 **	0.155	−0.209 *	−0.125	−0.156	0.001	−0.032	−0.266 **
LA (lumen area)	−0.062	0.158	−0.018	0.057	0.013	−0.01	−0.035	−0.126
LD (lumen diameter)	0.081	0.158	0.084	0.112	0.100	0.017	−0.05	0.043
**Doppler parameters**								
Frequency	0.025	−0.01	−0.193	−0.077	−0.149	−0.256 *	0.296 **	−0.01
PI (Pulsatility index)	−0.084	0.011	0.001	−0.118	0.014	0.163	−0.158	−0.159
RI (Resistive index)	−0.116	0.021	−0.033	−0.153	0.002	0.137	−0.128	−0.176

**Table 6:** Spearman’s rank correlation coefficient (Spearman’s ρ) between testicular size and echotexture parameters (evaluated by ultrasound-video analysis and ECOTEXT^®^ software) in *Rasa Aragonesa* rams (*n* = 96). Bold type indicates statistically significant correlations. * *p* < 0.05 and ** *p* < 0.01.

	Ec1 (Black Pixels)	Ec2 (White Pixels)	Ec3 (Grey Pixels)	TD (Tubular Density)	LA (lumen AREA)	LD (Lumen Diameter)
Scrotal circumference	−0.249 *	0.245 *	0.299 **	0.093	−0.227 *	−0.365 **
Testicular width	−0.188	0.145	0.171	0.118	−0.134	−0.245 *
Testicular length	−0.271 **	0.184	0.236 *	0.176	−0.210 *	−0.361 **
Testicular volume	−0.273 **	0.226 *	0.287 **	0.155	−0.225 *	−0.391 **

**Table 7:** Spearman’s rank correlation coefficient (Spearman’s ρ) between testicular size or echotexture parameters (evaluated by ultrasound-video analysis and ECOTEXT^®^ software) and testicular-artery blood flow (assessed by color-Doppler flow imaging) in *Rasa Aragonesa* rams (*n* = 96). Bold type indicates statistically significant correlations. * *p* < 0.05 and ** *p* < 0.01.

	Frequency	Pulsatility Index (PI)	Resistive Index (RI)
**Testicular size**			
Scrotal circumference	−0.067	−0.102	−0.111
Testicular width	0.160	−0.026	−0.024
Testicular length	0.041	0.083	0.109
Testicular volume	−0.037	−0.005	−0.002
**ECOTEXT^®^ parameters**			
Ec1 (black pixels)	−0.226 *	0.314 **	0.308 **
Ec2 (white pixels)	0.171	−0.422 **	−0.430 **
Ec3 (grey pixels)	0.179	−0.369 **	−0.371 **
TD (tubular density)	−0.120	0.490 **	0.520 **
LA (lumen area)	−0.211 *	0.386 **	0.387 **
LD (lumen diameter)	−0.192	0.225 *	0.218 *

## Data Availability

The data presented in this study are openly available in FigShare at https://doi.org/10.6084/m9.figshare.18102599 (accessed on 4 February 2022).

## Supplementary Materials

The following supporting information can be downloaded at: https://www.mdpi.com/article/10.3390/biology11020261/s1, Supplemental Table S1: Differences in testicular size, echotexture parameters (evaluated by ultrasound-video analysis and ECOTEXT^®^ software) and testicular-artery blood flow (assessed by color-Doppler flow imaging) between left and right testicles in *Rasa Aragonesa* rams. Results are shown as mean ± SEM of *n* = 96. Different letters indicate *p* < 0.05. Supplemental Figure S1: Monthly differences in testicular size (A), echotexture (C and D) and testicular-artery blood supply (B) in *Rasa Aragonesa* rams. Results are shown as mean ± SEM of *n* = 8. Different letters indicate *p* < 0.05. Supplemental Figure S2: Monthly differences in seminal quality parameters in Rasa Aragonesa rams. Results are shown as mean ± SEM of *n* = 8. Different letters indicate *p* < 0.05.
