# Supplementary material for: Testicular Ultrasound Analysis as a Predictive Tool of Ram Sperm Quality
Source: Biology (Basel). 2022 Feb 8;11(2):261. doi: 10.3390/biology11020261 (PMC8869597; doi:10.3390/biology11020261)
Supplement: Supplementary file 1 [file biology-11-00261-s001.zip › supplemental figures.pdf]

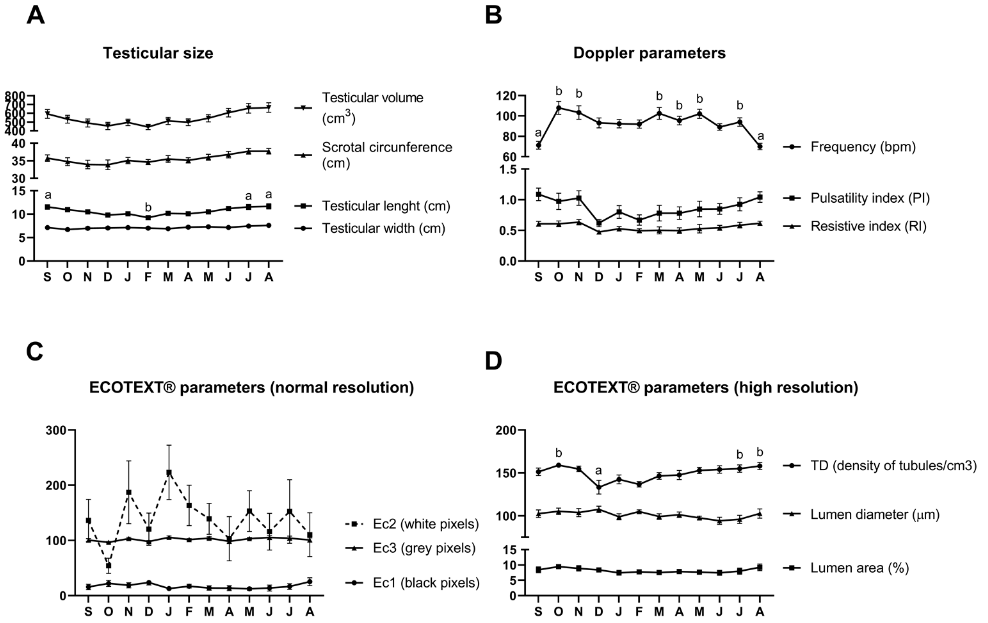

Figure S1: Monthly differences in testicular size (A), echotexture (C and D) and testicular-artery blood supply (B) in Rasa Aragonesa rams. Results are shown as mean ± SEM of n = 8. Different let-ters indicate $p < 0.05$.

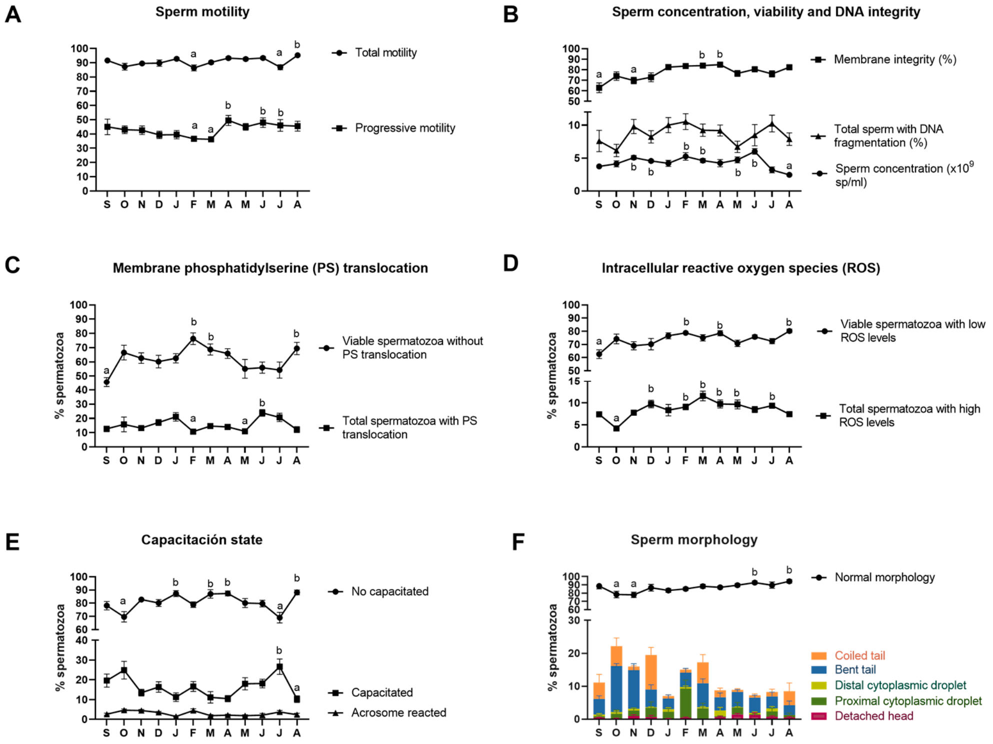

Figure S2: Monthly differences in seminal quality parameters in Rasa Aragonesa rams. Results are shown as mean ± SEM of n = 8. Different letters indicate $p < 0.05$.
