# Supplementary material for: Testicular Ultrasound Analysis as a Predictive Tool of Ram Sperm Quality
Source: Biology (Basel). 2022 Feb 8;11(2):261. doi: 10.3390/biology11020261 (PMC8869597; doi:10.3390/biology11020261)
Supplement: Supplementary file 1 [file biology-11-00261-s001.zip › Supplemental table S1.pdf]

Supplemental Table S1: Differences in testicular size, echotexture parameters (evaluated by ultrasound video-analysis and ECOTEXT® software) and testicular artery blood flow (assessed by Color-Doppler flow imaging) between left and right testicles in Rasa Aragonesa rams. Results are shown as mean ± SEM of n=96. Different letters indicate P<0.05.

	**Left testis**	**Right testis**
**Testicular size**		
Testicular width (cm)	7.11 ± 0.07	7.17 ± 0.06
Testicular length (cm)	10.69 ± 0.14^{a}	10.51 ± 0.14^{b}
**ECOTEXT® parameters**		
Ec1 (black pixels)	20.38 ± 2.18	18.43 ± 1.9
Ec2 (white pixels)	165.27 ± 16.55	184.23 ± 20.63
Ec3 (grey pixels)	101.61 ± 0.84	102.09 ± 0.95
TD (density of tubules/cm^3)	151.63 ± 1.53^{a}	147.04 ± 1.78^{b}
LA (lumen area, %)	8.31 ± 0.26	8.07 ± 0.24
LD (lumen diameter, μm)	100.87 ± 1.33	101.49 ± 1.32
**Doppler parameters**		
Frequency (bpm)	91.44 ± 1.77^{a}	94.08 ± 1.8^{b}
RI (Resistive index)	0.55 ± 0.01	0.56 ± 0.02
PI (Pulsatility index)	0.86 ± 0.03	0.88 ± 0.04
